# Long-term Survival in a Patient with Metastatic Spinal Cord Compression from a Prostate Cancer with Ultra-high PSA: Case Report and Review of the Literature

**DOI:** 10.7759/cureus.242

**Published:** 2015-01-22

**Authors:** Nhu Tram Nguyen, Sebastien Hotte, Ian Dayes

**Affiliations:** 1 Department of Radiation Oncology, Juravinski Cancer Centre-McMaster University; 2 Oncology, Division of Medical Oncology, Juravinski Cancer Centre-McMaster University; 3 Oncology, Division of Radiation Oncology, Juravinski Cancer Centre-McMaster University

**Keywords:** case report, literature review, prostate cancer, spinal cord compression, ultra-high psa

## Abstract

A 77-year-old man presented to the hospital for non-ambulation of 48 hours prior to admission.  He was found to have a metastatic spinal cord compression (MSCC), a PSA exceeding 27,000, and biopsy-confirmed prostate cancer. After palliative radiation (RT) to the spine and medical treatment, the patient recovered his functions fully and survived for more than 7.5 years, far beyond what would be expected based on current published literature.

A systematic review of the literature of MSCC in patients with prostate cancer was carried out. Prognostic factors of ambulation after RT included pre-treatment neurological status, duration of neurological deficits, and severity of the neurological impairment. Positive predictive factors of local control included single level of metastasis, time of development of motor deficits of more than 14 days, no prior androgen-deprivation therapy (ADT), age under 65, and longer course of RT (10 fractions of 2 Gy). Absence of prior ADT, pre-treatment ambulation, a single site of metastasis, and haemoglobin of less than 12g/L were positive predictors for survival.

## Introduction

Metastatic spinal cord compression (MSCC) occurs in 5-14% of patients with cancer during the course of their disease [1-2]. Although men with MSCC from prostate cancer fare better than patients with other malignancies, prognosis remains poor, with a median survival between 5.4 months and 1.71 year in non-surgical cohorts [3-6]. Prolonged survival and complete neurologic recovery in patients rendered paraplegic by MSCC is rare, especially in patients who have been paraplegic for longer than 24 hours prior to radiation therapy (RT).

Here is a unique case of a patient with Stage IV prostate adenocarcinoma who presented with diffuse vertebral body metastases and an initial prostate-specific antigen (PSA) in excess of 27,000, who recovered his functionality completely following MSCC and lived for more than seven years thereafter.

### Material/methods

The patient provided an informed consent for the publication of his case and his medical records were reviewed.

The English and French-language literature was reviewed according to PRISMA guidelines [7], using PubMed, Medline, Scopus, and OVID. The search term “prostate” was combined with “adenocarcinoma” or “cancer”, and “metastatic”, “cord compression”, or “spinal tumor”. All relevant abstracts and articles were thoroughly examined. Studies were included if they had:

1) patients with a biopsy-proven primary prostate cancer only,

2) documented radiological evidence of spinal cord compression,

3) patients treated with radiotherapy.

Studies were excluded if they 1) included patients with different types of primary cancers (not exclusively prostate cancers), 2) included patients treated surgically for their MSCC, 3) were abstracts or review articles, or 4) did not address patients’ outcomes after treatment of the spinal cord compression.

## Case presentation

Mr. GM, a 77-year-old man of Jamaican descent presented to hospital after 48 hours of inability of ambulation, weakness, numbness, and bilateral tingling in his legs. Review of systems revealed only a 14 lb. weight loss over two months. There was no history of trauma, sick contacts, or travels. His past medical history included benign prostatic hypertrophy, chronic kidney disease, and hypertension. His medications included Ramipril and Diclofenac. He was an ex-smoker (10 pack-year history).

On examination, the patient was bedbound. Vital signs were within normal limits (WNL). Neurological examination revealed strength in the lower extremities (LE) of 3+/5 for dorsi- and plantar flexion on the right leg and 4/5 on the left leg. The strength was 3/5 for the hip flexion bilaterally. The strength throughout the upper extremities (UE) was 5/5. Sensation to light touch and pinprick was normal for the UE, but diminished bilaterally in the LE without a clear sensory level. Deep tendon reflexes were 2+ for bilateral UE and knees; ankle jerks could not be elicited. Toes were downgoing on the left and upgoing on the right side. Cranial nerves, II-XII, were normal. The remainder of the examination was non-contributory.

Blood work showed a PSA of 27,394. Chest x-ray demonstrated bilateral pulmonary nodules and a loss of height in the C6-7 and T5 vertebral bodies. A MRI of the spine showed a 6 cm. soft tissue mass at the right lung apex, extending circumferentially and into the spinal canal, resulting in cord compression at T2-T4. There were diffuse bony metastases throughout the spine. A CT-guided biopsy of the lung mass revealed metastatic prostate carcinoma. A prostate biopsy showed prostate adenocarcinoma, Gleason 9 (4+5). Staging abdomino-pelvic CT images showed a large pelvic mass involving the bladder, seminal vesicles, rectosigmoid, and retroperitoneal adenopathy. The bone scan showed diffuse bony metastases.

### Treatment

The patient received one dose of dexamethasone 10 mg. IV followed by emergent RT. The radiation was delivered to C7-T5 vertebral bodies and the right upper lung mass with cobalt, using a parallel opposed pair (POP) arrangement. He received 20 Gy in five fractions; dexamethasone, 4 mg QID, that was tapered off. Bicalutamide, 50 mg PO daily (for four weeks), and goserelin, 10.8 mg SQ, were administered; goserelin was continued as trimestrial treatments. One week after initiation of treatments, PSA was 27,300 and three weeks later, it was 9,180.

A few months later, the patient was fully ambulating with a walker and independent for his daily activities. After two years of goserelin, the patient was ambulating without assistance and his PSA was 18. One year later, it rose to 38; CT scan showed progressive hepatic metastases; all the adenopathy had regressed. Bicalutamide, 50 mg PO daily, was initiated, and his PSA decreased to 11. The PSA rose again after a year, and leuprolide was initiated. After not responding to intermittent bicalutamide, this was switched to nilutamide. His disease progressed, and he died 7.62 years after his initial presentation with MSCC.

## Discussion

Prostate cancer is the second leading cause of MSCC in men, after lung malignancies. Because patients with prostate cancer may live long enough to develop a recurrence of MSCC, functional recovery, local control (LC) in the treated spinal cord region, and even overall survival are of particular importance in this population.

### PSA at presentation and response to treatment

This patient presented with a MSCC and an initial PSA of 27,394, which is, to our knowledge, the highest ever reported PSA level in a patient. Surprisingly, the PSA only decreased to 27,300 after one week of treatment and then to 9,180 three weeks after. This is most likely related to the high dose “Hook effect” [8]. This phenomenon occurs when high concentrations of an analyte give an artifactually lower value when measured by sandwich assays [9]. Vaidya, et al. [8] have advocated the use of a two-step procedure to overcome the effect of excess antigen where unbound excess antigen is washed away before adding the signal antibody to form the sandwich. In fact, this two-step procedure has since been implemented in our institution, but was not routinely performed when this patient first presented.

### Review of the literature

This patient had a Tomita score of Grade 3 (i.e., he was non-ambulatory) for 48 hours prior to admission, and his Harrington’s classification was Class III [10-11]. Both are significant adverse prognostic factors. Furthermore, a PSA of >40 ng/mL predicts worse outcome [12], which was also the case for our patient. Thus, his functional outcome and longevity were both remarkable given the gravity of his symptoms and his elevated PSA at initial presentation. We have conducted a literature review regarding prognostic factors for ambulation, local control, and survival in patients with prostate cancer who undergo RT for a MSCC. The results of our search are listed in Table 1*.*

**Table 1:** Review of the literature of patients with prostate cancer treated with radiotherapy for a metastatic spinal cord compression and their outcome after treatments. PCa: prostate cancer; ADT: androgen deprivation therapy; MSCC: metastatic spinal cord compression; RP: radical prostatectomy; RT: radiotherapy; S: surgery; MS: median survival; LC: local control; #: fraction; d: days P: prospective study, R: retrospective review; +: positive predictive factors; (-): negative predictive factors

Author	Type of study	n	Patients characteristics	Diagnostic modality	Treatment for PCa prior to MSCC	Treatments of MSCC	Outcomes	Prognostic Factors
Huddart [4]	R	69	58% non ambulant 52% catheterised. 19% had MSCC as initial diagnosis	Myelography (42%) MRI (47%)	ADT (25%)	20 Gy/5# (14%) 28-30 Gy/9-10# (54%) 35-40 Gy/15-20# (14%) S (20%) Also dexamethasone + ADT.	52% had improvement of motor power 63% regained ambulation Among patients who improved, 77% did so within 7 days. MS 115d (5-2016 days). MS if no prior ADT: 627 d (46-1516 d).	+ functional recovery: single level, no prior ADT, age <65 years >30 Gy or S did not affect outcome. + for OS: single site, Hb >12g
Rades [16]	R, multicenter study	281	57% ambulatory, 43% >14d of motor deficits	CT or MRI	ADT (87%)	8 Gy/1# to 20 Gy/4# vs 30-40 Gy/10-20#	Response to RT 86%. 33% regained ability to walk.	+ for LC : time developing motor deficits < 14d, single site, RT schedule, long course RT
Nagata [5]	R	26	100% paraplegic/quadriplegic from MSCC	MRI myelography	steroids, ADT, RT +/- S, none (42%)	20-39 Gy/10-20#	58% paraplagic despite ADT 8% remission of paralysis.	
Tazi [6]	R	24	13% ambulant with mild neurological deficits 59% parapetic 38% paraplegic	Myelography, MRI, CT-scan	RP (4%), ADT (66.7%), bilateral orchidectomy.(33.3%)	RT alone (50%) RT-S (38%), castration alone (4%), steroids only (8.3%) 20 Gy/5# to 30 Gy/10#	63% ambulant after treatment. 89% S-RT were ambulatory vs 58% RT alone. MS=4 mos	
Kuban [17]	R	41		Myelography, Bone scan, CT scan, XR	S	S-RT or RT-S (17%) RT alone: 25-40/10-20# (83%)	46% OS < 6 mos 20% < 2 mos 5% had recurrence within irradiated field 2% had recurrence at margin of previous iradiated field MS =4 mos.	
Aass [18]	P	49				Median RT dose 30/10# (9-40 Gy/3-20#)	MS= 3.5 months (range 0.3–36.0).	No predictive factors of outcome : time from diagnosis to start of RT, histology, stage at diagnosis, time from symptoms of MSCC to start of RT, age, number of lesions causing MSCC, pre-treatment function.
Smith [14]	R	35	34% ambulatory 34% parapetic 14% paraplegic	Myelogram and/or MRI		RT, steroids and ADT S also in 9%	100% remained ambulatory. 83% who were paraparetic regained ambulation. However, 20% of these patients had recurrent compression and became paraplegic. 80% with paraplegia remained paraplegic despite treatment. Overall, 27% had recurrent compression. Mean OS of paraplegic patitents (3.9 mos) worse than whole group (18 mos)	
Zelefsky [13]	R	42		Complete myelography		(Dexamethasone 100 mg, then 24 mg QID x 3 days then tapered, RT) Median RT dose: 30 Gy/10#		+ for OS: ambulatory status, CR on follow-up myelography. Extent of MSCC and anatomic location has no effect.

Factors predicting neurological recovery after RT are summarized in Table 2. Patients with bony compression have worse outcomes than those without bony compression. In Zelefsky’s review, the chance of reopening a complete myelographic obstruction associated with a compression fracture was 11% compared to 50% for patients without bony compression (p=0.007) [13].

**Table 2:** Prognostic factors of functional recovery in patients with prostate cancer treated for a metastatic spinal cord compression with radiotherapy. RT: radiotherapy

Positive prognostic factors of ambulation	Not prognostic of ambulation
Degree of neurological damage pre-RT	RT schedule
No bony compression	
Neurological symptoms > 14 days prior to RT > 8-14 days> 1-7 days	
Improvement of power by Day 7 post-RT	
Age < 65 years old	
Single level of compression	
No prior androgen deprivation therapy	

Huddart, et al. also suggested that the time of treatment from the onset of severe neurological deterioration might be more crucial than the time from the first neurological symptoms [4]. Finally, the strongest prognostic factor for functional recovery remains the degree of neurological damage before treatment [6]. Smith, et al. reported that 92% of ambulatory patients, 83% of paraparetic patients, and 20% of paraplegic patients were ambulatory after appropriate treatment [14]. Similar trends were observed in other studies [4, 6, 15]. 

Predictive factors of LC are shown in Table 3. The two-year LC of MSCC reported by Rades, et al. was 84% [16]. Huddart, et al. reported an actuarial three-year risk of further episodes of MSCC at the initial or adjacent site of 50% [4].

**Table 3:** Predictive factors of local control

Positive predictive factors of local control
Absence of prior androgen deprivation therapy
Radiotherapy schedule
Time of development of motor deficits of more than 14 days
Single level metastasis
Age less than 65 years old

In most studies, median survival of patients with no previous history of prostate cancer was 5.4 months to 3.5 years (range: 46 days to 4.15 years) [3-6].

Factors associated with improved survival following RT for MSCC are summarized in Table 4. Patients without prior ADT had significantly longer survival than those with prior ADT, with a median survival of 15.5 months compared to six months [4, 6]. Pre-treatment ambulatory status and degree of response have also been correlated with improved survival. In Zelefsky’s study, the median survival was 9.5 months among patients who obtained a complete response compared to to months for those who did not respond [13].

**Table 4:** Prognostic factors of survival in patients with prostate cancer and treated for a metastatic spinal cord compression with radiotherapy. PSA: prostate-specific antigen

Positive prognostic factors of prolonged survival	Not prognostic of survival
Absence of prior androgen deprivation therapy	Gleason score
Pre-treatment ambulatory status	Total number of metastases
Degree of response post-treatment documented by repeat myelography (complete response better than partial response, which is in turn better than no response)	Degree of spinal cord compression
Single site metastasis	Ultra-high level of PSA
Haemoglobin >12 g/L	

## Conclusions

In summary, sustained function after more than 24 hours of non-ambulation, prolonged LC, and long survival is rare in patients with MSCC. This is even rarer in the context of diffuse bony and vertebral body metastases. Our patient had improvement of function, for more than 7.5 years, after a very poor pre-treatment neurologic status with an initial 48 hours of paraplegia. Although he was hormone naïve, the severity of his clinical presentation, his exceedingly high PSA, age over 75 years, and diffuse vertebral bony metastases, there is no clear explanation for his tremendous functional recovery and long survival, both exceeding far beyond that expected from current published literature. To our knowledge, this is the first documented case of survival of over 7.5 years in a male patient over the age of 75 with widely metastatic prostate adenocarcinoma with a MSCC and a PSA in excess of 27,000.
